# Biobased Functional Carbon Materials: Production, Characterization, and Applications—A Review

**DOI:** 10.3390/ma11091568

**Published:** 2018-08-31

**Authors:** Catalina Rodriguez Correa, Andrea Kruse

**Affiliations:** Department of Conversion Technologies of Biobased Resources, Institute of Agricultural Engineering, University of Hohenheim, Garbenstrasse 9, 70599 Stuttgart, Germany; Andrea_Kruse@uni-hohenheim.de

**Keywords:** biomass, carbon materials, pyrolysis, hydrothermal carbonization, activation, water treatment, energy storage, gas separation, adsorption

## Abstract

Even though research on porous carbon materials from biomass dates back to at least hundred years, it is still an extremely relevant topic. These materials can be found in applications that range from those that are widely known, such as water treatment, to others that are newer and indispensable for the transition towards environmentally friendly technologies, such as lithium- and sodium-ion batteries. This review summarizes some of the most relevant research that has been published concerning production technologies, insights on the chemical reaction mechanisms, characterization techniques, as well as some examples of the applications and the properties that the carbon materials must fulfil to be used in those applications.

## 1. Introduction

Biomass is a term frequently used for plants or plant-derived materials. However, biomass encompasses a wider range of materials from biological origin. According to the Renewable Energy Directive of the European Union, biomass is defined as “*the biodegradable fraction of products, waste and residues from biological origin from agriculture (including vegetal and animal substances), forestry and related industries including fisheries and aquaculture, as well as the biodegradable fraction of industrial and municipal waste*” [1]. Many types of agricultural residues are grass or wood-like, thus they can be classified as lignocellulosic biomass. Lignocellulosic biomass is the most commonly studied, which consists of cellulose, hemicellulose, lignin, extractives, moisture, and inorganics. In the past decades, biomass has received increasing attention as an energy and carbon source due to climate change, the necessity to reduce CO_2_ emissions, and the depletion of fossil sources. 

A considerably large amount of research has been dedicated to biomass for energy applications (e.g., combustion or gasification), recovering platform chemicals, and for char production, a carbon-rich solid material used for e.g., soil amending. Another important research field for biomass is the production of porous materials like activated carbon, intended for processes where adsorption plays a major role. Even though the term “adsorption” was first proposed by J.W. Gibbs in the late 1800s, the earliest applications of char as an adsorbent date back to Hippocrates and are related to the relieving of stomach ailments [2]. Charcoal’s adsorptive properties were studied for the first time in 1773 by Swedish scientist Carl W. Scheele, who measured the gas adsorption capacity of charcoal to determine adsorption forces [3]. Johann Tobias Lowitz discovered the adsorptive properties of charcoal in liquid media by removing the oily, brown-colored substance (rich in *phlogiston*) from tartaric acid [4,5]. He also used charcoal to purify oils, vinegar, and alcoholic beverages, among others. The findings from Lowitz lead to the development of carbon adsorbents for sugar discoloration purposes in the beginning of the 19th century [6]. One of the most relevant applications was in gas masks for protection against mustard gas in the trench warfare during World War I [7]. Nowadays, there are innumerable applications where activated carbon is utilized; therefore, exploring and understanding the use of renewable and environmentally friendly precursors such as biomass to produce these materials, gives an important impulse towards the development of a sustainable bio-economy.

Activated carbon is a very versatile material used to purify, deodorize, decolorize or separate and recover different components [8]. It is also found in energy storage systems (lithium-ion batteries, supercapacitors), and in catalysts or catalyst support [9]. The flexibility of activated carbon is given by its textural properties and by the possibility of tailoring the surface chemistry based on a targeted application. Activated carbon production consists of a two-step process: first the precursor (i.e., biomass) is carbonized typically via pyrolysis at high temperatures in an inert atmosphere, which leads to the formation of a carbon-rich and thermally stable product (pyrochar) [10]. Recently, hydrothermal carbonization (HTC) has become an interesting process to thermochemically convert biomass, especially wet biomass, into a carbon-rich product (hydrochar) [11]. For this reason, it has also been explored as a carbonization step prior to activation. After the carbonization process, the hydro- or pyrochars undergo an activation process to increase the surface area. This occurs at high temperatures and in the presence of a physical (water steam or CO_2_) or chemical (KOH, H_3_PO_4_, ZnCl_2_, NaOH, etc.) activating agent. Nowadays, most of the activated carbon available is produced by means of physical activation; a considerably smaller fraction is produced by means of chemical activation [12]. Concerning the precursors, activated carbons are primarily obtained from wood and coal (bituminous, anthracite, lignite), or, to lower extent, by peat and coconut shells. Coconut shells have become increasingly relevant in the activated carbon production chain and companies are investing more in using them as precursor, due to their renewable character and the fact that the products obtained have similar properties to those obtained from coal. Both wood and coconut shells are hard, lignin-rich biomasses with low ash content. Other hard biomasses such as almond shells and cherry or apricots stones have also been tested in a laboratory scale and they presented similar or superior properties to those presented by coconut shells. On the other hand, several studies have shown that soft biomass with a lower lignin content is also suitable to produce activated carbon with the same properties. The constant price increase of fossil coal, together with the fact that the market is focusing only on coconut shells as alternative renewable precursor to produce activated carbon, has led to an increase of 8–5% of the activated carbon price [13].

Carbon is a very versatile element due to its different hybridization states, which give rise to different physical and chemical properties. Carbon can be organized in a crystalline structure (e.g., graphite or diamond), can form tubes, or can be stacked in disorganized graphene sheets to build amorphous carbon. Depending on the precursor composition and on the processing parameters, it is possible to obtain graphite (graphitizing or non-graphitizing carbon). The first one to report this phenomenon was Rosalind E. Franklin, who stated that parent materials with a high hydrogen content allowed the formation of graphite since hydrogen content allowed “*the continued formation of hydrocarbon decomposition products in the interior of the structure during the early stages of carbonization,* [which] *prevents the carbon from ‘setting’ at very low temperatures*” [14]. On the other hand, a large oxygen content in the precursor is the cause for the formation of a non-graphitizing carbon.

The carbons obtained from biomass are typically non-graphitizable amorphous carbons and depending on the biomass composition, the carbons have different chemical and structural properties. Activated carbon is a carbon-rich porous material that can be obtained by converting biomass with a two-step process consisting of carbonization and activation [15]. During the carbonization process, the biomass components undergo different decomposition reactions at different temperatures, leading to different carbon-rich materials with different properties and a high thermal stability. During the activation step, the carbon rich material is oxidized to develop the surface area and to form functional groups on the surface [16]. The surface area, pore size distribution and surface chemistry depend on the temperatures and reaction times of both steps as well as on the type of activating agent. During a physical activation process, the precursor is carbonized at high temperatures in an oxygen-depleted atmosphere, followed by an activation process using an oxygen-rich activating agent such as water steam, CO_2_, or air. When acidic or basic substances are used in the activation step, the process is known as chemical activation [17]. 

## 2. Carbonization Technologies

### 2.1. Pyrolysis

Pyrolysis is the thermochemical decomposition of carbonaceous substances in an inert atmosphere and at high temperatures. The main products are a carbon-rich solid (pyrochar), a liquid phase composed of water soluble organic compounds and tar, and a mixture of permanent gases. The distribution of the product yields is strongly dependent on the heating rate and final temperature. If the desired product is pyrochar (which is the case of bio-based carbon materials), the heating rate must be slow, and the reaction temperature should be higher than the temperature at which the organic fraction of the precursor has completely converted into char. In the case of biomass, this temperature is usually above 550 °C. Below this temperature, the solid product will contain a fraction of unconverted cellulose (or even hemicellulose if the reaction temperature is considerably low). This means that the solid product will have a relatively high oxygen and hydrogen content, and it will be thermally unstable. 

During the decomposition of lignocellulosic biomass, hemicellulose, cellulose and lignin are cleaved into volatile compounds composed mostly of oxygen, hydrogen, and nitrogen. The decomposition degree of biomass is directly dependent on temperature: at low temperatures (approx. 150 °C) lignin decomposition begins, albeit at very slow rates, and it continues over a wide temperature range (over 800 °C) with increasing rates. The reason for this is the different chemical bonds, and thus binding energies that constitute lignin (especially carbon-carbon bonds that need high cleavage temperatures). At around 180 °C, hemicellulose decomposition begins, closely followed by cellulose (at approximately 260 °C). These two polysaccharides decompose over a smaller temperature range (up to 400 °C) and at considerably higher decomposition rates than lignin, because carbohydrates are macromolecules built-up by only one type of chemical bond, i.e., identical chemical binding energies [18].After biomass is pyrolyzed at temperatures close to 550 °C, the char obtained is composed of a carbon matrix and a low content of organic substances (remaining undecomposed lignin fraction) [19]. When the char is exposed at temperatures higher than 550 °C, not only is the remaining organic fraction converted into char, but the carbon matrix also undergoes an aromatization process that increases its crystallinity degree and thermal stability. Above 800 °C, the amorphous nature of char starts transforming into a graphitic-like structure. Yet, since lignocellulosic char is non-graphitizable (i.e., it cannot be converted into graphite solely by heat treatment at temperatures larger than 3000 °C and atmospheric pressure [20]), a fully crystalline structure cannot be achieved [21,22,23].

The stoichiometric decomposition of cellulose after reaching thermodynamic equilibrium can be described with Reaction (1):

(1) $$C_{6}H_{10}O_{5}\to 3.74C+2.65H_{2}O+1.17{\mathrm{CO}}_{2}+1.08{\mathrm{CH}}_{4}$$

This amount of carbon can be regarded as the theoretical maximum amount of carbon that can be obtained. M. J. Antal et al. determined that high pressures and a closed vessel are paramount to obtain materials with this carbon content [24,25,26]. This is because secondary reactions between carbon-rich volatile compounds are favored. According to the reaction mechanism proposed by Banyasz et al., char is almost exclusively a secondary product from the interaction between tars at high temperatures, since the direct char formation (cellulose → anhydrocellulose → char) plays an unimportant role in these conditions [27]. Consequently, if secondary reactions are favored at high pressures, the carbon yield increases. Contrarily, if thermodynamic equilibrium is not reached (which is usually the case), the solid product has a higher carbon content than the precursor but is lower from the theoretical value. The incomplete cellulose decomposition in Reaction (2) was proposed by Klason et al. after describing the product from the carbonization of cellulose at 400 °C [28]:

(2) $$8C_{6}H_{10}O_{5}\to C_{30}H_{18}O_{4}+23H_{2}O+4{\mathrm{CO}}_{2}+2\mathrm{CO}+C_{12}H_{16}O_{3}$$

Another interesting property of pyrolysis char is its relatively large porosity and surface area, which is the result of the volatilization of organics and it is directly dependent on the temperature and the heating rate [29]. The largest surface areas attainable occur at temperatures between 600–800 °C, since most of the volatiles have been removed [21]. However, these areas are not large enough to be employed in technologically relevant applications, such as gas separation techniques or energy storage systems, where surface areas larger than 800 m^2^/g are desired [30]. To achieve this, a secondary step, namely activation, is necessary.

### 2.2. Hydrothermal Carbonization (HTC)

Hydrothermal carbonization (HTC) is a technology that was developed in 1913 by the German scientist Friedrich Bergius while studying coal formation processes [31]. This thermochemical conversion process has received increasing attention in the past decade, since it is an energetically more efficient way than pyrolysis to carbonize wet biomass. The reason is that water is used as a reaction medium (and as a reactant). Consequently, water must not be evaporated before the decomposition reactions. This process has been used to convert sewage sludge [32], biogas digestate [33] and animal manure [34]. It is also a better carbonization process for biomass with a small particle size (e.g., wood sawdust) compared to pyrolysis, since pyrolysis of small particulate material leads to low char yields [35].

As opposed to pyrolysis, biomass decomposes incompletely during HTC and the products are a solid carbon-rich material (hydrochar), a gas phase composed mainly of CO_2_, water and water-soluble compounds. At hydrothermal conditions, mainly the carbohydrates in biomass are involved in the formation of hydrochar (Reaction (3)). Hemicellulose and cellulose are hydrolyzed into simple sugars, which in turn are converted into hydroxymethylfurfural (HMF) via dehydration reaction [36]. Subsequently, HMF can follow two paths: either it is decomposed into formic and levulinic acid or it polymerizes to form hydrochar via water elimination, aldol condensation and Diels-Alder addition reactions [37]. There is clear evidence that supports the catalytic effect of protons on the HMF production [38]. On the other hand, it has also been shown that very low pHs shift the reaction towards the formation of levulinic acid as a secondary product from the HMF decomposition [39]. This could be a disadvantage in terms of hydrochar production, since hydrochar is the product of HMF polymerization. Concerning lignin, its chemical structure is modified due to partial hydrolysis [40]. This can be seen from the high concentration of phenolic compounds in the liquid phase (although it is important to bear in mind that some aromatic compounds can also come from short-chain intermediates [41]). However, lignin decomposition is not as strong as that of cellulose and hemicellulose. This was observed after comparing the thermal decomposition and the FTIR spectra of different lignins and lignin-rich biomasses with the respective hydrochars [42]. Additionally, since lignin has a shielding effect on the carbohydrates, biomasses with a high lignin content lead to hydrochars that are thermally less stable than biomasses with a low lignin content or than model substances such as glucose, fructose or holocellulose [43].

(3) $$C_{6}H_{10}O_{5}\to C_{5.25}H_{4}O_{0.5}+3H_{2}O+0.75{\mathrm{CO}}_{2}$$

Hydrochars have a lower carbonization degree compared to the chars obtained from pyrolysis. This indicates a lower crosslinking and a lower polymerization degree and, consequently a low thermal stability. On the other hand, hydrochars have higher oxygen and hydrogen contents than pyrochars, hence they have a richer surface chemistry. Concerning morphology, hydrochar is usually a powder with a small particle size (in the case of hydrochar from sugars, the particles are agglomerates constituted of 2–10 µm microspheres [44]), whereas pyrochar usually retains the structure of the parent material. This is a consequence of swelling and disintegrating of the biomass particles during HTC. However, hydrochar properties can change noticeably if exposed to higher temperatures. R.K. Garlapalli et al. pyrolyzed hydrochars from digestate at different temperatures and compared them with pyrolyzed digestate. The pyrolyzed hydrochars had similar carbon contents to the pyrolyzed digestate but the ash content was lower. Additionally, the oxygen content was higher, which could indicate a richer surface chemistry. The hydrochar pyrolysis also led to a larger surface area [45]. This was also evidenced by X. Zhu et al. after converting *Salix psammophila* wood and other biomasses into char via HTC and pyrolysis [46].

Being a powder, hydrochar is an interesting precursor to produce powder activated carbon, since it makes up to 48% of the market [3]. Some applications for powder-activated carbons are electrodes for energy storage systems, water treatment (e.g., decolorizing agent), air purification (e.g., mercury removal), as well as the food and pharmaceutical industry. They are also used for the production of catalysts; one common example is palladium-coated activated carbon powder for hydrogenation reactions [47].

## 3. Activation Processes

The activation process can be regarded as a partial gasification of the carbon matrix of the precursor. This can occur either physically, employing water steam or CO_2_ as activating agents, or chemically, making use of chemicals such as KOH, H_3_PO_4_, K_2_CO_3_, NaOH, ZnCl_2_, among others. Physical activation processes generally make use of carbon-rich precursors (e.g., fossil coal, peat or biochar) or lignocellulosic biomass. The reaction takes place at temperatures high enough so that the Boudouard, the water-gas shift, and other gasification reactions (Reactions (4)–(6)) can occur [2].

*Boudouard reaction*

(4) $$C+{\mathrm{CO}}_{2}⇄2\mathrm{CO}$$

*Water-steam gasification*

(5) $$C+H_{2}O⇄\mathrm{CO}+H_{2}$$

*Water-gas shift reaction*

(6) $${\mathrm{CO}}_{2}+H_{2}⇄\mathrm{CO}+H_{2}O$$

On an industrial scale, the most common physical activation processes are conducted with water steam. A disadvantage of physical activation processes is the relatively low yields due to carbon loss as volatile matter prompted by the gasification reactions. An alternative is chemical activation processes. These can be carried out either with carbon-rich precursors (char or fossil coals) or with biomass. Promising results were also obtained using non-conventional precursors like plastics, old tires or sewage sludge, which lead to activated carbons with surface areas as high as 2800 m^2^/g [48]. Nonetheless, biomass is the most desired precursor in view of the development of a bio-based economy. The activation process can be conducted in one or two steps; during the two-step activation, the precursor is first carbonized in the absence of oxygen and then the char is impregnated with the activating agent. This is followed by a heating process at temperatures usually below 800 °C. The one-step activation omits the carbonization step. During chemical activation, the reaction temperatures are lower than those needed for physical activation. Chemical activating agents act as catalysts of the gasification reactions; therefore, these reactions can occur at lower temperatures and the yields are higher. One drawback of chemical activation processes is the acidic or basic nature of the activating agent, which can lead to serious corrosion problems; some are hazardous for the environment as well as for human health [49]. 

Activated carbons obtained chemically usually have micropore volumes than those obtained physically [16,50]. H_3_PO_4_ is the most common activating agent for the industrial production of activated carbon, since low temperatures (around 450 °C) are needed to obtain a considerably large microporosity. After impregnation with H_3_PO_4_ and application of heat, the biomass particles swells and the organic fraction decomposition is catalyzed by the acid, which leads to porosity development [51]. A downside of activating with H_3_PO_4_ is the importance that phosphor plays in agricultural practices and the fact that there are limited phosphate reserves [52]. For this reason, arguments like the “food vs. fuel” dilemma can be debated. An alternative for H_3_PO_4_ can be KOH or any other alkali metal compound. KOH has already been tested on an industrial scale and activated carbons with a surface area close to 3000 m^2^/g (theoretical maximum for graphene [53]) have been obtained [54]. Alkali metals act as gasification catalysts, forming lamellar structures with the carbon that are then destroyed, which explains the pore formation [55]. Besides corrosion problems and toxicity, another drawback of using KOH is that only activated carbons in powder form can be obtained. Even though there are several applications where powder activated carbons can be found, their use in applications where pellets or granules are necessary (e.g., gas separation or gas storage) is limited.

### 3.1. Chemical Activation with KOH

Potassium formed from KOH has been the preferred activating agent to produce microporous carbon materials, since it leads to large volumes of micro- and ultra-micropores. The first attempt to understand the chemical reaction between potassium and carbon was in the context of coal gasification. Potassium acts as a catalyst that promotes the formation of CO_2_ and CO; hence, it can be considered a gasification catalyst. The catalytic behavior of potassium has been explained usually parting from potassium carbonate in the presence of CO_2_ and H_2_O, as stated by Reactions (7)–(9) and Reactions (10)–(12), respectively [56,57].

*C-CO_2_ reaction system*

(7) $$\;K_{2}{\mathrm{CO}\;}_{3}+2C\to 2K+3\mathrm{CO}$$

(8) $$\;K_{2}{\mathrm{CO}\;}_{3}+2C\to 2K+3\mathrm{CO}$$

(9) $$\;K_{2}O+{\mathrm{CO}\;}_{2}\to K_{2}{\mathrm{CO}}_{3}$$

*C-H_2_O reaction system*

(10) $$\;K_{2}{\mathrm{CO}\;}_{3}+2C\to 2K+3\mathrm{CO}$$

(11) $$\;2K+2H_{2}O\to 2\mathrm{KOH}+H_{2}\;$$

(12) $$\;2\mathrm{KOH}+\mathrm{CO}\to K_{2}{\mathrm{CO}\;}_{3}+H_{2}$$

C. Spiro and D. McKee [58] proposed a reaction mechanism where KOH is an intermediate product from the reaction between K_2_CO_3_, C, and a CH group, which is assumed as a moiety. This mechanism can be adapted for KOH as follows:

(13)  KOH+C→KH+CO 

(14) $$\;\mathrm{KH}+\mathrm{CH}\to K+C+H_{2}\;$$

(15) $$\;\mathrm{KH}+\mathrm{KH}\to 2K+H_{2}\;$$

In this scenario, Reactions (14) and (15) indicate the reaction termination. 

These mechanisms have been studied experimentally and through modelling to determine parameters like reaction rates, activation energy, temperature dependence, and standard free enthalpies, among others, albeit only in relation to coal gasification processes [57,59,60,61,62]. To understand the differences between chemical activation with alkali metals and an alkali-catalyzed gasification, several groups use temperature programmed desorption (TPD to track the products from the reaction. M. A. Lillo-Ródenas et al. observed a reaction between KOH and an anthracite coal over a wide temperature range and measured large H_2_ concentrations at temperatures as low as 400 °C. In addition, they measured significant CO and CO_2_ concentrations only at temperatures higher than 800 °C, which they interpreted as the decomposition of carbonates formed from the hydroxide and the coal. The formation of the carbonates was corroborated via FTIR measurements [63,64]. Based on these experiments, M. A. Lillo-Ródenas et al. proposed one of the most widespread mechanisms that describe the reaction taking place during chemical activation with KOH (Reaction (16) [63]. Nevertheless, this reaction should be used cautiously, especially when transferring it to the chemical activation process of biomass or chars/coals with high oxygen content. Firstly, one of the products is metallic potassium, which is extremely reactive in the presence of water; secondly, thermodynamic calculations indicate that this reaction is only possible at temperatures higher than approximately 630 °C, but in reality, biomass char can be activated at lower temperatures. The reason for this is the high reactivity of low-rank coals or chars compared to graphite [42,64].

(16) $$\;6\mathrm{KOH}+2C\to 2K+2K_{2}{\mathrm{CO}\;}_{3}+3H_{2}$$

Another mechanism that is frequently mentioned in the literature is the formation of lamellar structures composed by carbon and potassium ions. W. Wen [55] explained these compounds as Electron Donor-Acceptor complexes and explained its formation through a cyclic process based on the mechanism proposed by D. McKee et al. [56]. Reactions (17)–(20) correspond to the reaction with CO_2_ and Reactions (21)–(24) to H_2_O: 

*C-CO_2_ reaction system*

(17) $$\;K_{2}{\mathrm{CO}\;}_{3}+2C\to 2K+3\mathrm{CO}$$

(18) $$\;2K+2\mathrm{nC}\to 2C_{n}K\;$$

(19) $$\;2C_{n}K+{\mathrm{CO}\;}_{2}⇄2C_{n}K\cdot \mathrm{OCO}⇄\left(2\mathrm{nC}\right)\cdot K_{2}O+\mathrm{CO}$$

(20) $$\;\left(2\mathrm{nC}\;\right)\cdot K_{2}O\;+{\;\mathrm{CO}}_{2}⇄\left(2\mathrm{nC}\right)\cdot K_{2}{\mathrm{CO}}_{3}⇄2\mathrm{nC}+K_{2}{\mathrm{CO}}_{3}$$

*C-H_2_O reaction system*

(21) $$\;K_{2}{\mathrm{CO}\;}_{3}+2C\to 2K+3\mathrm{CO}$$

(22) $$\;2K+2\mathrm{nC}\to 2C_{n}K\;$$

(23) $$\;2C_{n}K+H_{2}O⇄2\mathrm{nC}+2\mathrm{KOH}+H_{2}\;$$

(24) $$\;2\mathrm{KOH}+\mathrm{CO}⇄K_{2}{\mathrm{CO}\;}_{3}+H_{2}$$

In both scenarios, the stoichiometric composition of the lamellar compounds is directly related to the temperature (Table 1).

If this temperature dependence is true, this could explain the high surfaces from the MAXSORB activated carbons [54]. These carbons were activated with a two-step heating ramp: first, the mixture of precursor and KOH was heated up to 400 °C, where it was left for two hours. This was followed by another heating step up to 600–900 °C. It is possible that during that first step, many of the intercalate compounds of the form C_24_K were formed, explaining the high surface area and microporosity of the product. J. Alcañiz-Monge et al. observed the chemical activation process of different coals by means of thermogravimetric analysis and TPD. Their results showed the same behavior as the results from M.A. Lillo-Ródenas et al. [63], since during the decomposition, H_2_ was measured at low temperatures and the decomposition rate was related to the reactivity of the coals. However, stoichiometric calculations concerning the yield and surface area showed that porosity development is not only a result of gasification reactions, but also of the aforementioned intercalation compounds [65].

Although it is well known that KOH and K_2_CO_3_ are corrosive and difficult to work with at high temperatures, a clear majority of studies on chemical activation with potassium compounds focus on them as activating agents. There are some experiments that concentrate on other potassium salts such as K_3_PO_4_ [66], KCl [66], KNO_3_ [66], and potassium oxalate [67]. J. Laine and A. Calafat [66] showed that KNO_3_, KOH, K_2_CO_3_ and K_3_PO_4_ lead to the high surface areas in that order after activating at 800 °C under a CO_2_ atmosphere for 1 h. They also used KCl as activating agent, but the surface area obtained was even lower than when no activating agent was used. Similar results were obtained by W. Tsai et al. [68] following the same procedure as J. Laine and A. Calafat. In both works, activated carbons were produced via a one-step activation, i.e., no carbonization step was conducted. J. Hayashi et al. [69] compared KOH and K_2_CO_3_ as activating agents to produce activated carbon at different temperatures under N_2_ for 1 h. They found that KOH was a better activating agent than K_2_CO_3_ at lower temperatures, leading to higher surfaces areas. This tendency was inverted at higher temperatures, where K_2_CO_3_ was responsible for the highest surface areas. It is common to find in the literature that the best temperature to obtain high surfaces with K_2_CO_3_ and KOH is around 800 °C, which is close to the melting point of K_2_CO_3_ (891 °C); however, this temperature is considerably higher than that of KOH (360 °C) [70,71,72]. There are several studies involving different reaction parameters, yet there is no thorough study that analyzes the chemical and physical properties of the different potassium salts in relation to the activated carbon properties.

### 3.2. Effect of the Carbonization Process on the Structural Properties of Microporous Carbon Materials

As was previously mentioned, chemical activation can be done by following either a one-step or two-step process. The one-step process parts from the direct impregnation or dry mixing of the precursor with the activating agent, followed by a heating process up to the activation temperature and a subsequent washing and drying steps to remove the activating agent as well as the sub-products formed during the activation from the product. The two-step activation process involves a carbonization step prior to the impregnation and heating steps. Variables such as temperature, time, KOH concentration and N_2_ flow rate have been modified to understand their influence on the textural and adsorption properties. Yet, in the case of a two-step activation process, there is still not enough information about the influence of the carbonized precursor properties on the activated carbon textural characteristics and adsorption parameters.

The findings of M. Lillo-Ródenas et al. [73] show that pyrolyzing biomass prior to a chemical activation undermines the development of the surface area. However, it was determined by C. Rodriguez Correa et al. that a carbonization step is necessary to obtain high yields and large surface areas [18]. Here, the surface areas were considerably larger than those presented by L. Khezami et al. [74]. The difference between both works was the pyrolysis temperature. L. Khezami used a higher temperature for the pyrolysis than for the activation. This means that the activation precursor (i.e., the pyrolysis char) was thermally very stable. The results presented by C. Rodriguez Correa et al. were obtained by pyrolyzing and activating at the same temperature; therefore, the precursor was thermally unstable to some extent during the activation. This was also observed by M. Evans et al. [72], who used pyrolyzed spent coffee grounds and macadamia nut shells at 750 °C as carbonized precursor for a chemical activation at 750 °C. Clearly, the thermal stability of the precursor is important to develop large surface areas as well as high yields; nevertheless, it is still not clear what the interrelation between the carbonization (first step) and the activation (second step) temperatures during a two-step activation process is.

Another interesting aspect is the link between the chemical composition of the char and the structural and chemical properties of the activated carbon. C. Rodriguez Correa et al. observed that using hydrochars and pyrochars for activation led to similar total surface areas and to a similar distribution of acidic surface functionalities [42,75]. Yet, neither the basic groups nor the crystallinity degree were studied. In scientific literature, it can be found that activated carbons from biomass have an amphoteric character and can be acidic or basic in an aqueous solution. Additionally, the minerals inherently present in biomass influence also the total pH of the surface. Studies have also shown that minerals are leached from the char after a KOH activation. Nevertheless, the ash content of the char is not 0% [33,42]. Accordingly, the effect of the remaining minerals on the surface pH should be studied further. 

The crystalline structure of activated carbons is an important property that directly influences other properties; it is also relevant for other applications. One example is electrical conductivity, which is essential in energy storage systems or fuel cells [76]. Rosalind E. Franklin stated in her work that precursors with a very high oxygen content lead to non-graphitizable carbons, which is the case of biomass. On the other hand, precursors with a high H content can lead to graphitizable carbons [14]. If biomass is converted by means of HTC, the hydrochars obtained have a higher C and a lower O content than biomass, but it also has higher O and H contents than pyrochars. Thus, it would be of special interest to study the crystalline structure of hydrochars after exposing them to a second heat treatment like pyrolysis or activation.

## 4. Characterization of Activated Carbons

Activated carbons are materials widely employed in applications involving sorption processes. Sorption is the general term used to describe the interaction between a substance (adsorbate) and the surface of a porous solid (adsorbent). The most common sorption mechanisms are absorption and adsorption. Adsorption is the general term that refers to separation processes that occur in the interface between the adsorbent and a compound present in a liquid solution or gas mixture (adsorbate). Adsorption is ruled by steric (pore diameter vs. molecular size; the adsorbate molecules must be small enough to enter in the pores of the adsorbent), equilibrium (adsorption capacity; the adsorbent must be able to accommodate the adsorbate on the surface), and kinetic (the adsorbate is transported from the bulk solution to the adsorbent surface due to a concentration gradient) driven mechanisms. Adsorption can be regarded as a thermodynamic driven process described by the variation of the potential energy of the system as a function of the distance between the adsorbate particles and the adsorbent surface. The potential energy of the system is affected by the properties of the adsorbent (e.g., surface structure, chemical groups and impurities on the surface), adsorbate (e.g., chemical nature and orientation of the molecule when approaching the surface) and by their interaction. The adsorbate-adsorbent interaction can be electrostatic attraction due to intermolecular forces like van der Waals (physisorption), or it can be a chemical bond with the superficial functional groups (chemisorption). These processes are not mutually exclusive and can occur simultaneously, depending on the solid. 

According to the theory proposed by Langmuir, during physisorption, the adsorbate molecules strike against the surface and they are held by van der Waals forces until they evaporate again (desorption). The system potential during physisorption is a function of the distance between the centers of the interacting species *r* and is ruled by attraction forces *Φ_A_* that result from dispersion forces (Equation (25)) and by repulsion forces *Φ_R_* (Equation (26)).

(25) $$\Phi_{A}=-\frac{A_{1}}{r^{6}}-\frac{A_{2}}{r^{8}}-\frac{A_{3}}{r^{10}},$$

(26) $$\Phi_{R}=\frac{B}{r^{12,}}$$

where *A_x_* and *B* are constants.

The first term in Equation (26) is always dominant and is related to dipole-dipole interactions; the second and third terms correspond to dipole-quadrupole and quadrupole-quadrupole interactions, respectively. If only the dominant term in Equation (26) is regarded, the physisorption potential of the total system can be described by Equation (27), which is analogous to the Lennard-Jones potential (Equation (28)).

(27) $$\Phi_{Physi}=\frac{B}{r^{12}}-\frac{A_{1}}{r^{6}},$$

(28) $$\Phi_{LJ}=4\epsilon \left[{\left(\frac{\sigma }{r}\right)}^{12}-{\left(\frac{\sigma }{r}\right)}^{6}\right],$$

**Figure 1:**
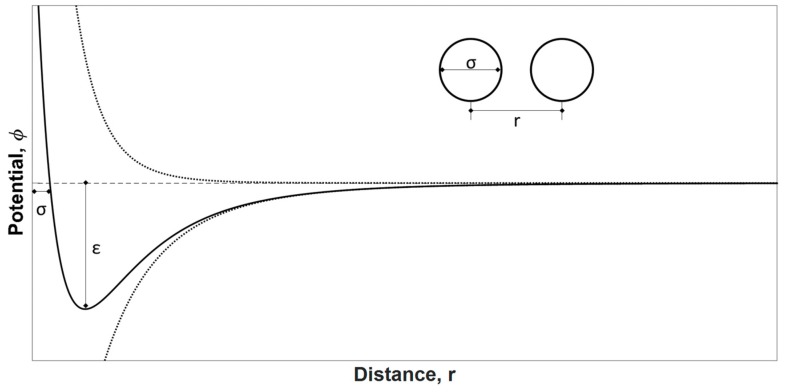
Graphic representation of the Lennard-Jones potential (See similar figures in textbooks of physical chemistry).

The force constants *ε* and *σ* are specific to each molecule and are available for many common species. Figure 1 depicts a graphic representation of the Lennard-Jones potential.

The energy released during physisorption is relatively small (same order of magnitude as the condensation enthalpy); therefore, the residence time of the molecules on the surface at ambient conditions can be very short. This means that the adsorption process is completely reversible and fast. Additionally, during physisorption, molecules can organize on the surface as a monolayer or they can build multilayers; however, the surface is not chemically modified since there are no electron transfer processes involved. Chemisorption, on the other hand, is a usually non-reversible process and the energies involved are considerably larger (related typically to covalent bonds). Consequently, the residence times on the surface are longer. During chemisorption, molecules on the surface of the adsorbent can only be organized in a monolayer. In addition, the chemical nature of the surface changes because of electron transfer processes, i.e., formation of surface functional groups. The total system potential of chemisorption is not only ruled by attraction and repulsion forces, but also electrostatic energies, which must be taken into consideration (e.g., polarization).

To characterize porous solids, the parameters that are most frequently measured are the textural properties (surface area and pore size distribution), surface chemistry, and adsorption properties on the medium where the process will be carried out (gas or liquid).

### 4.1. Textural Characterization

**Figure 2:**
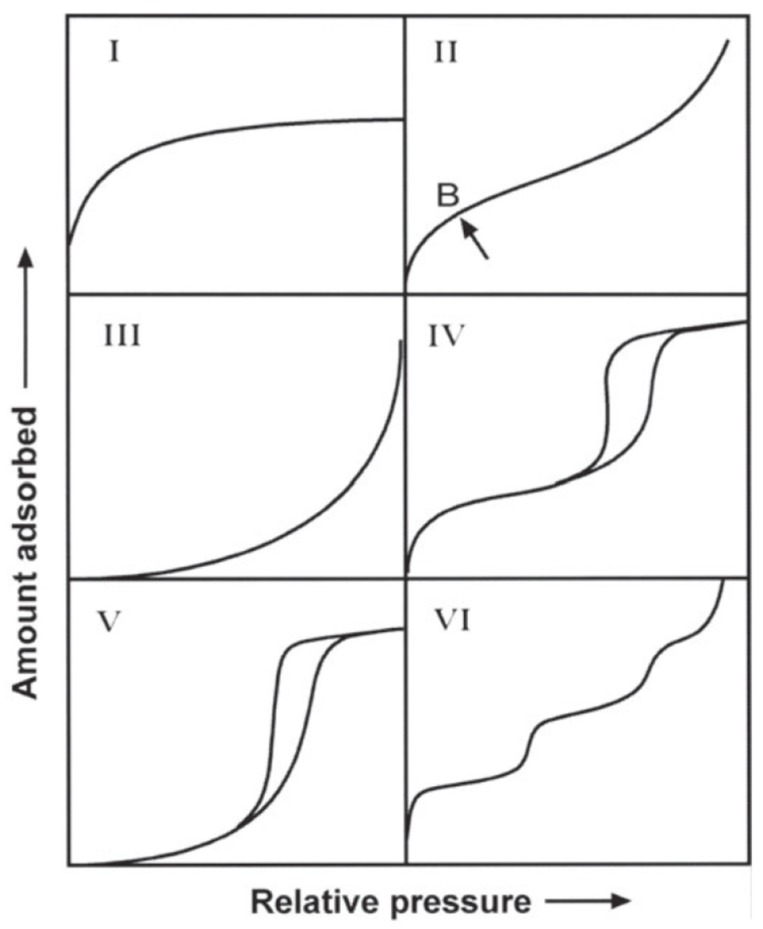
IUPAC classification for gas isotherms. Type I is typical of highly-microporous solids; type II for non-porous or microporous solids; type III reveals a weak or no interaction between adsorbent and adsorbate or a strong interaction between adsorbate molecules; type IV is typical for mesoporous solids and the hysteresis indicates condensation; type V indicates pore condensation and weak interaction; type VI corresponds to a stepwise multilayer adsorption on a non-porous surface (special case) (Reprinted from [77] with permission from Springer Nature).

The total surface area of a solid is comprised by its internal (wall area of pores) and external surfaces. There are several techniques available to determine the surface area of solid. Some of them are mercury intrusion porosimetry, small angle x-ray scattering, scanning electron microscopy, and gas adsorption (physisorption). Gas adsorption is one of the most popular methods since it is straightforward, simple and not so cost-intensive. It also comprises of a wide range of pore sizes (from 0.35 nm up to > 100 nm) [77]. To determine surface areas and pore size distributions, gas isotherms are measured at pressures below the vapor pressure of the chosen gas. These isotherms are classified in six categories according to the IUPAC and are characteristic of the textural properties of the solid (Figure 2). The most common gases employed are N_2_, CO_2_, Ar and Kr, and depending on the gas properties at the measuring temperature, different properties from the porous solid can be assessed in more detail. Typical measuring conditions and applications for these gases are presented in Table 2.

The gas isotherm itself is not enough to determine the surface area, since the number of molecules covering the surface (monolayer) must be known. There are several models available to describe the isotherms based on kinetic or thermodynamic approaches. An example of the kinetic approach is the Langmuir model, which is the basis for many newer and more complex models; the Langmuir model was proposed based on many assumptions. The most important ones are: (i) adsorption occurs only in a monolayer, (ii) there are no interactions between adjacent adsorbed species, and (iii) the surface is perfectly smooth and homogeneous. The Niemark-Kiselev model is a thermodynamic approach. The Brunauer-Emmett-Teller (BET) model [79] is an expansion of the Langmuir model, and is one of the most commonly employed models, since it describes accurately the formation of the monolayer based on probabilities (Equation (29)). It assumes that the Langmuir equation can be applied for every adsorption layer, i.e., it includes multilayer adsorption. The model states that high-energy sites on the adsorbent surface (e.g., narrow pores, edges or heteroatoms) will be covered first by the adsorbate, since in these sites there is an overlap of the interaction potentials. The monolayer acts as an adsorption site for the second layer, the second for the third, and so on. However, this model considers that only the initial monolayer is adsorbed on the surface by means of an induced dipole. In turn, the subsequent multilayers are adsorbed by means of the same forces acting in condensed vapors, i.e., they have the same properties as the liquid state [80]. The BET surface area is frequently calculated from the linear part of the graph, comprised between the relative pressure range of 0.05 and 0.35. At lower relative pressures, the surface heterogeneity plays a significant role (pore sizes, functional groups and drastic adsorption heat differences between one part of the surface and another), and at higher relative pressures, adsorption by capillary condensation becomes relevant.

(29) $$\frac{p}{n\left(p^{0}-p\right)}=\frac{1}{n_{m}C}+\frac{C-1}{n_{m}C}\left(\frac{p}{p^{0}}\right),$$

The monolayer capacity, *n_m_*, can be calculated from the BET model (Equation (29)), where *n* is the number of moles adsorbed at a pressure *p*, *p* is the pressure of the system after equilibrium is reached, *p*^0^ is the vapor pressure of the adsorbate, and *C* is the BET constant, which must always be larger than zero. With this information, the specific surface area can be calculated using Equation (30), where *N_A_* is the Avogadro constant, *A_x_* is the area occupied by one molecule of adsorbate (cross sectional area; Table 2), and *M_AC_* is the sample weight of the activated carbon.

(30) $$S_{BET}=\frac{n_{m}N_{A}A_{x}}{M_{AC}},$$

The application of the BET model for activated carbons has been highly questioned because adsorption takes place through micropore filling, instead of a monolayer formation. Additionally, the BET model assumes that every molecule belonging to any layer above the monolayer contributes all its full latent heat of liquefaction, regardless of the number of neighbors surrounding it. However, this can only happen if the coordination number is 12, which does not necessarily happen. For this reason, the latent heat released is usually lower [80]. To address this problem, J. Rouquerol et al. [81] proposed the following considerations:

1. The quantity *C* should be positive (i.e., a negative intercept on the ordinate of the BET plot is the first indication that one is outside the appropriate range).
2. Application of the BET equation should be restricted to the range where the term *p*/[n*(*p*_0_ − *p*)] continuously increases with *p*/*p*_0_.
3. The *p*/*p*_0_ value corresponding to nm should be within the selected BET range.

Although calculating the BET surface area from the nitrogen adsorption isotherm is a common procedure, it can lead to large deviations from the “real surface area”, especially if the material is highly microporous. The main reasons are the low measurement temperature and the interaction of the N_2_ quadrupole with the solid surface. At 77 K, the diffusion of N_2_ molecules inside the smaller pores is kinetically restricted, especially at lower relative pressures, making it difficult to reach equilibrium during the isotherm measurement [78]. Furthermore, the inherent quadrupole causes a strong interaction between superficial functional groups and the N_2_ molecule. This translates into the modification of the monolayer and of the micropore filling pressure [82]. A way to overcome this problem is to measure gas isotherms with Ar at 87 K or CO_2_ at 273 K. Measurements with CO_2_ are convenient since its molecular dimensions are very similar to N_2_ and the diffusion kinetics are improved, since the isotherm is measured at significantly higher temperatures than with N_2_ [83]. Besides, no expensive equipment is needed to set the temperature (water with ice suffices). CO_2_ isotherms are particularly interesting for the characterization of microporous and ultra-microporous materials, since narrow microporosity can be determined. At 273 K, the saturation pressure of CO_2_ is approximately 3.5 MPa and the highest pressure that most of the available equipment can achieve is 0.1 MPa. This means that the pores determined by this measurement are usually <1 nm (larger micropores are not included). Rodríguez-Reinoso et al. made a comparison that included more than 100 different activated carbons from lignocellulosic material and he concluded that carbons can be classified into three groups based on the micropore volumes obtained from N_2_ and CO_2_ isotherms [84,85]:

- V_N2_ < V_CO2_: Activated carbons with very low burn-off (<5%). The differences are attributed to the restricted diffusion of N_2_. CO_2_ can penetrate very narrow microporosity and/or there are constrictions in the entry of the micropores.
- V_N2_ ≈ V_CO2_: Activated carbons with low-to-medium (<35%) burn-off. The microporosity is relatively narrow and homogeneous.
- V_N2_ > V_CO2_: Activated carbons with medium-to-high burn-off. The microporosity is wider and very heterogeneous.

Despite the high temperatures used to measure CO_2_ isotherms, kinetic limitations due to constrictions and narrow pores should not be excluded [86]. Measurements with Ar at 87 K lead to more reliable results, due to the lack of a quadrupole or a dipole. Consequently, Ar exhibits no interaction with superficial groups. In addition, given that the measurement temperature is slightly higher than with N_2_, diffusion occurs faster [87]. On the other hand, measuring with Ar can be considerably cost-intensive, since setting the experimental temperature requires either liquid argon or special equipment. 

**Figure 3:**
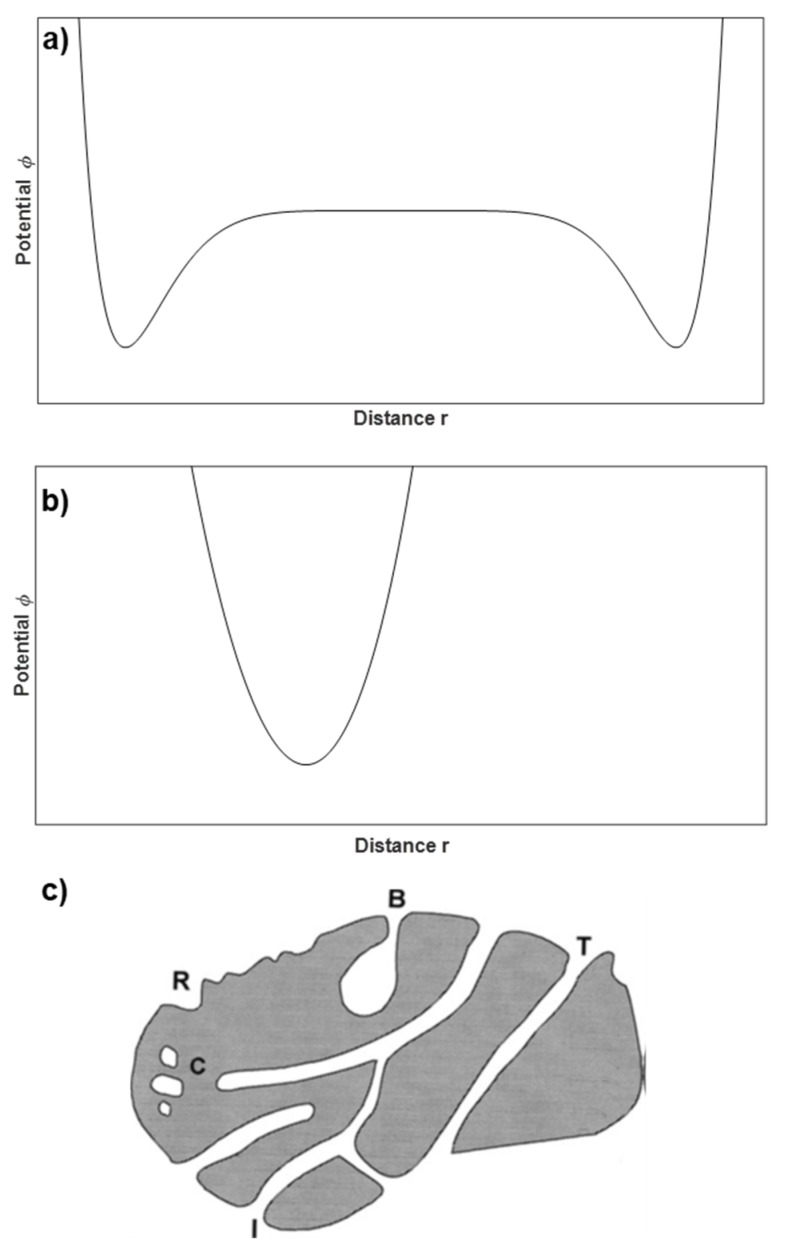
Adsorption potentials (**a**) inside a mesopore, (**b**) inside a micropore, and (**c**) cross section of a hypothetical porous grain showing various types of pores: closed (C), blind (B), through (T), interconnected (I), together with some roughness (R) (Adapted from [88] with permission from UNESCO).

Pore size distribution is another important parameter that can be derived from the different gas isotherms. The IUPAC classifies pores as macropores (>500 Å), mesopores (20–500 Å), and micropores (<20 Å). Likewise, micropores are divided into two categories: super-micropores (7–20 Å) and ultra-micropores (<7 Å). The filling of the pores is a continuous process and strongly depends on the interaction potential between the solid and the adsorbate as well as on the pore dimensions and shape [77]. Mesopores are usually filled following the capillary condensation mechanism: at relatively low pressures (*p*/*p*^0^ < 0.1) the monolayer is formed, followed by the formation of multilayers at higher pressures. When the multilayers have reached a critical thickness and the relative pressure is high enough (*p*/*p*^0^ > 0.7), capillary condensation occurs. The explanation for this mechanism is that inside the pore, there are two independent potentials due to the large distance between the walls (Figure 3a). These materials usual show type IV isotherms (Figure 2) and the isotherm hysteresis is defined by the pore geometry. Regarding micropores, it is widely accepted that the filling mechanism is not capillary condensation, but a different type of pore-filling mechanism. The main reason is that the pore size has usually the same dimensions of one adsorbate molecule and the pore walls are so close together that instead of presenting two separate potentials, they merge together resulting in a considerably larger potential (Figure 3b). Highly microporous solids present adsorption isotherms type I and the different hypothetical pore shapes are described in Figure 3c. 

Based on empirical, macroscopic and thermodynamic approaches as well as using molecular simulation, models have been developed to describe microporosity and pore size distribution from these isotherms. One of the first and more fundamental models is the *t*-plot proposed by B.C. Lippens and J.H. de Boer [89,90]. This empirical model was developed to calculate the specific surface area of a porous solid and it uses a reference curve obtained from a non-porous material with a similar BET *C* constant. The *t*-curve is a plot of volume of gas adsorbed vs. the standard multilayer thickness of the reference non-porous material, given by Equation (31):

(31) $$t=\frac{n}{n_{m}}d^{\prime },$$

where *n* is the number of moles adsorbed, *n_m_* is the molar capacity of the monolayer and *d’* is the effective thickness of the monolayer (*d’_N_*_2_ = 0.354 nm). For porous materials, the standard multilayer thickness is described by several authors, but the most common one is the equation obtained by de Boer [91] (Equation (32)):

(32) $$t={\left(\frac{13.99}{log\left(p^{0}/p\right)+0.034}\right)}^{\frac{1}{2}}\;Å,$$

For carbon materials, the statistical layer thickness is calculated with Equation (33) (ASTM D6556-1 Standard Test Method for Carbon Black—Total and External Surface Area by Nitrogen Adsorption).

(33) $$t=0.088{\left(\frac{p}{p^{0}}\right)}^{2}+0.645\left(\frac{p}{p^{0}}\right)+0.298\;\mathrm{for}\;p/p^{0}=0.2-0.5$$

From the *t*-plot and making use of the specific surface area obtained from the BET model, the external and microporous surface areas can be calculated by following Equation (34):

(34) $$S_{micro}=S_{BET}-\left(\frac{V_{liquid}}{t}\right)\times 10^{4},$$

where *S_micro_* is the microporous surface area, *S_BET_* is the BET surface area and *V_liquid_*/*t* × 10^4^ is the external surface area; *V_liquid_* is the adsorbed volume expressed as the corresponding liquid volume (*V_liquid_ =* 15.47 *x V_adsorbed_*(*STP*) for N_2_ at 77 K) and *t* is the thickness of the standard multilayer at the end of the *t*-plot.

The empirical model proposed by Dubinin and Radushkevich (DR), which was later expanded by Dubinin and Astakhov (DA; Equation (35)), is based on the potential theory of Polanyi [92] and on the fact that the adsorbate that fills the pores has the same properties as the liquid state [93]. Consequently, the concept of specific surface area loses its physical sense and is no longer considered. The DR model considers that an adsorbed molecule is trapped between the surface (maximum potential) and the layer, where the adsorption potential is zero. The assumptions for this model state that the adsorption potential is independent of the amount of adsorbate on the surface and from the temperature, and that, at equal conditions, the interaction between the adsorbed molecules is the same as that between the adsorbate molecules that are not adsorbed.

(35) $$V_{adsorbed}=V_{0}exp\left[-{\left(\frac{RT}{\beta E}\;ln(p^{0}/p)\right)}^{m}\right],$$

where *V_adsorbed_* is the volume adsorbed at the relative pressure *p*/*p*^0^, *V*_0_ is the micropore volume, *R* is the ideal gas constant, *T* is temperature, *E* is the adsorption energy, *β* is the affinity coefficient of the adsorbate and *m* is a constant related to the homogeneity of the pore size distribution. In the case of the DR model, *m* = 2, indicating a homogeneous pore size distribution. This model has been strongly criticized, since it is only valid for very low pressures (*p*/*p*^0^ < 0.2 for activated carbons), which can be difficult to achieve for N_2_ isotherms. Alternatively, CO_2_ isotherms can be used as a complement to the N_2_ isotherms since the vapor pressure of CO_2_ at 273 K is higher than that of N_2_ at 77 K. Thus, the relative pressure range that can be covered accounts for partial pressures up to 0.3 and the micropore range is fully described.

Molecular simulation methods like the grand canonical Monte Carlo, quantum chemical calculations or density functional theory (DFT) describe the adsorption in porous solids based on statistical mechanics and thermodynamics of nanophases. DFT was developed by N.A. Seaton et al. [94] to calculate the pore size distribution by disregarding effects such as capillary condensation, which are not valid for very small pores. They proposed that a measured adsorption isotherm is the sum of the isotherms of each individual pore. This is valid for porous solids with an internal surface area considerably larger than the external surface. This means that the total number of moles adsorbed *N* at a certain pressure *p* is the integral of the isotherms (expressed in terms the molar density *ρ* of the adsorbate at a pressure *p* inside a pore with a size *w*), multiplied by the pore size distribution *f*(*w*) (Equation (36)):

(36) $$N\left(p\right)=\int_{w_{min}}^{w_{max}}f\left(w\right)\rho \left(p,w\right)dw,$$

This model comprises of the complete range of micropore-mesopore sizes; however, it is valid only for liquid-liquid interactions and cannot describe fluids close to the solid surface. For this reason, the DFT model was expanded to the non-local DFT (NLDFT), which includes fluid-fluid and fluid-solid interactions. The NLDFT model includes simple pore geometries (slits, cylinders and spheres), but it assumes that the pore walls are smooth, which can lead to inconsistencies. A newer model, the quenched-solid DFT (QSDFT), was proposed to include surface rugosity and energetic heterogeneities [95]. 

### 4.2. Surface Chemistry Characterization

Graphite is a crystal made from graphene sheets organized following a stacking sequence *ababab*. In this crystal, three of the four carbon electrons form regular covalent bonds with neighboring atoms (σ-electrons) and the fourth electron resonates between valence-bonds (π-electron). The free mobility of the π-electron is directly related to the electrical properties of graphite. However, this occurs only within the basal plane since the graphene layers are stalked together due to van der Waals forces, which explains the anisotropic character of graphite. Bio-based microporous materials possess microcrystallites of graphene sheets; however, the graphene sheets are randomly oriented (turbostratic) and their content and size depend on the production process [76,96].

**Figure 4:**
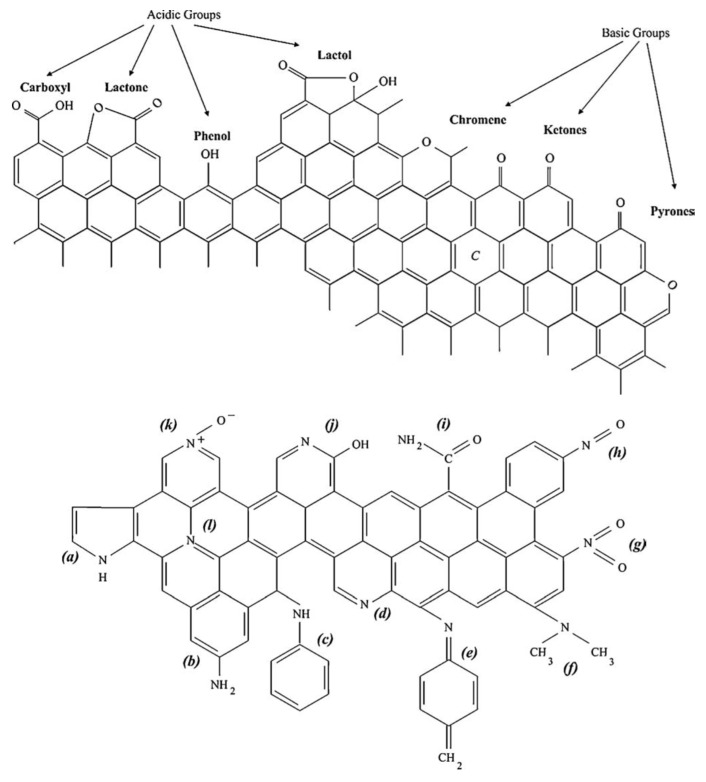
Functional groups containing oxygen (top; from ref. [102] with permission from Elsevier) and nitrogen (bottom; from ref. [103] with permission from Elsevier).

The microcrystalline structure of bio-based carbon materials is not continuous and crystallographic defects such as layer edges, structural carbon vacancies, non-aromatic rings or dislocations are of major importance. Vacancies, for example, do not only disturb the crystalline pattern but also can be filled with impurities. Edge dislocations determine the connection between microcrystallites, preventing the right orientation and thus the *ababab* stacking sequence. Lattice defects are also the sites with higher densities of unpaired electrons [97]; hence, they are active sites for the bonding of functional groups containing heteroatoms. These groups influence adsorption processes since they determine factors such as wettability, electrical and catalytic properties, as well as the possibility to further modify the surface with new groups. Oxygen is by far the most influencing heteroatom and, unlike nitrogen, it binds to the carbon atom spontaneously even at low temperatures and low partial pressures to form either acidic (e.g., carboxyl, hydroxyl, lactones, or lactol groups) or basic groups (e.g., chromenes, pyrones, or quinones), as shown in Figure 4. It should be noted that non-heteroatomic sites characterized by regions with a high density of π electron within the basal planes are also considered as basic centers, but of the Lewis type, and it is believed that it is the most common type of basic sites [98]. 

Nitrogen-containing groups (Figure 4, bottom) are also of considerable importance but they are not formed as effortlessly as oxygen-containing groups. Nitrogen functional groups can only be formed if the precursor is rich in structural nitrogen (e.g., chitin and chitosan) [99] or through additional reactions with nitrogen-containing reagents [100]. Contrary to the oxygen groups, the classification of the acidic or basic character of the nitrogen groups is not as straightforward, since it depends on the heterogeneity of the different groups created. Nonetheless, some generalizations can be made: formation of groups at low temperatures with nitrogen-containing reagents leads to the formation of slight acidic groups such as lactams, imides and amides. On the other hand, heat treatments at high temperatures lead to the formation of pyridines and pyrrolic structures, which have a basic character [97].

Hydrogen carbon complexes, sulfur, phosphorous, halogens and boron also form surface functionalities but the information about their properties is relatively scarce. On the other hand, it is known that they affect the total carbon pH and, thus, adsorption and catalytic processes. Carbon-hydrogen complexes, for example, are bonded considerably stronger than carbon-oxygen and usually occurs at the edge of the crystals of graphite or in the interior of the char particles. It is also known that the hydrogen content affects directly the electrical resistivity of carbon blacks (carbon black is a colloidal product obtained from a controlled thermal decomposition of gaseous hydrocarbons and is composed of almost pure carbon. It should not be confused with soot—sometimes also referred to as black carbon, which is a particulate carbon material produced from different thermochemical processes) [97,101]. Phosphorous groups are known to work as fire retardant and oxidation protectors of the carbon and they usually appear after a chemical activation with H_3_PO_4_ [51]. Sulfur containing groups can be formed after a reaction with oxygen-containing groups or by addition to unsaturated sites

There are several techniques that allow the characterization of surface functionalities. Spectroscopic techniques comprise of methods such as Raman spectroscopy, X-Ray diffraction (*XRD*), nuclear magnetic resonance (*NMR*), and Fourier Transformed Infrared Spectroscopy (*FTIR*). FTIR is commonly used as a qualitative technique to describe the type of groups present on the surface. This technique takes advantage of the changes in the electric dipole moment that molecules experience during expansion, contraction, or bending vibrations. Some surface groups have assigned IR absorption bands, making their identification possible; however, there are certain groups that are assigned to the same range of absorption bands (e.g., the range 1585–1600 cm^−1^ has been assigned to carbonates and to the stretching of the C=C bonds in aromatic rings), which causes difficulty in interpretation of the results. Another downside of *FTIR* measurements is that particle light-scattering occurs, which causes the baseline to shift in the high frequency region. Moisture and grinding conditions can also affect the spectrum. Even though *FTIR* is a qualitative method, semi-quantitative analysis of modification treatments can be conducted by defining ratios of integrated absorbance [104]. 

To quantify the surface functionalities, techniques such as Boehm titration, temperature programmed desorption (*TPD*) or the point of zero charge (*pH_PZC_*) are better suited than FTIR. Boehm Titration is a method developed by the German scientist H. P. Boehm [105] to analyze the oxygen-containing surface groups of carbon black particles and it has been successfully employed for the characterization of activated carbons [106], carbonized aerogels [107], and biochars [108]. The oxygen-containing acidic groups usually behave as Brønsted acids, donating protons to water molecules and, hence, negatively charging the carbon surface. Contrarily, the basic groups accept protons, which positively charges the surface. The acidic groups are detected by allowing the char to react with bases from different strengths (NaOH, Na_2_CO_3_, NaHCO_3_ and NaOC_2_H_5_) and the basic groups are determined with HCl. The total consumption of NaOH provides information on the total number of acidic groups on the surface. Considering that Na_2_CO_3_ neutralizes carboxylic and lactonic groups, and NaHCO_3_ neutralizes carboxylic acids, these groups can be quantified. In addition, by subtracting the consumption of Na_2_CO_3_ and NaHCO_3_ from the NaOH consumption, hydroxylic groups can be determined. The consumption of sodium ethoxide gives information on extremely weak acids (e.g., carbonyl groups); however, it is not so widely used because the experiment must be conducted in a non-aqueous solution and in the total absence of oxygen [97,105]. The drawback of Boehm titration is that it is sensitive to surface topography. For example, longer equilibration times might be needed for highly porous carbons than for carbons with a lower surface area to determine all the functional groups. Furthermore, restricted access to pores influences the reactant consumption and the presence of hydrophilic groups that interact with water can affect the result. Finally, it should be considered that the Boehm titration assumes that all acidic groups are oxygen-containing functionalities in the form of carboxylic, lactonic, and hydroxyl groups. It does not take into consideration the other types of oxygen-containing groups or that some groups involve other type of heteroatoms. The *pH_PZC_* is frequently determined by titrating a solution containing the carbon material at different pH values. Yet, there are no standard methods or norms, making it difficult to compare different data from the literature. 

### 4.3. Adsorption in Gaseous Media 

Gas adsorption is one of the most common techniques to characterize the surface of activated carbons; however, measuring gas isotherms is not a trivial task. The amount of gas adsorbed on the surface at a constant temperature can be determined either volumetrically or gravimetrically. The gravimetric method is convenient for measurements close to room temperature and it is relatively straightforward: mass changes are recorded with a microbalance and the gas amount is controlled with a pressure gauge, but a pressure dependence buoyancy correction needs to be considered. The volumetric method, on the other hand, is useful for measurements at cryogenic temperatures. With this method, the amount adsorbed cannot be determined directly. Instead, the excess amount adsorbed (i.e., the amount of gas put in contact with the adsorbent minus the amount that is not adsorbed) is measured. For this, the exact void volume must be known [77].

**Figure 5:**
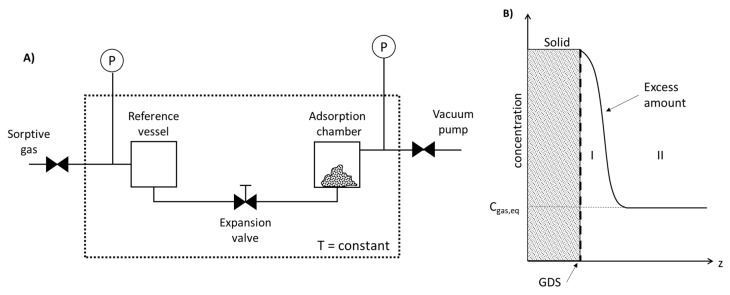
(**A**) Scheme of the experimental setup for a volumetric measurement, (**B**) Gibbs representation of the adsorbed amount on a solid (adapted from [109] with permission from Elsevier).

During the volumetric method, a sorptive gas is expanded into a vessel containing the adsorbent at constant temperature (Figure 5A), where part of the gas is adsorbed by the adsorbent and part remains in the vessel. The volume adsorbed *V_adsorbed_* can be calculated by multiplying the surface area of the porous solid *S* times the adsorption layer thickness *t* and the adsorbed amount from the gas concentration *n_adsorbed_* can be calculated if the void volume *V_void_* is known (Equations (37) and (38))

(37) $$V_{adsorbed}=At,$$

(38) $$n_{total}=n_{adsorbed}+n_{remain}=A\int_{0}^{t}Cdz+\int_{0}^{V_{void}}C_{gas,eq}dV,$$

However, one of the most relevant difficulties of this method is establishing the boundary between the adsorbed phase and the bulk gas phase to determine adsorption layer thickness. Therefore, in the late 1800s J.W. Gibbs introduced the concepts dividing surface (GDS) and excess amount adsorbed. The dividing surface is an imaginary surface defined parallel and closely to the actual surface of the solid. By establishing this, the uncertainty of the surface location was solved. Figure 5B represents the concentration gradient as a function of the distance. By assuming that there is no absorption, the concentration inside the solid is 0. At the GDS, the adsorbed amount is the highest and it decreases with the distance (zone I) until the distance from the solid is large enough so that the concentration is the same as the equilibrium concentration of the vessel (zone II) [110]. The excess amount adsorbed *n_excess_* is a necessary concept that results from the difficulty to differentiate between the bulk gas phase and the layer. It can be graphically understood as the part of the curve that is above the rectangle formed by the x-axis and the line representing the equilibrium concentration of the gas. At very low temperatures or pressures, it can be assumed that *n_excess_* = *n_adsorbed_* since the concentration of the gas in the bulk phase, *C_gas,eq_,* is relatively low. At higher temperatures and pressures, *C_gas,eq_* is considerably larger and it influences the adsorbed amount (Equation (39)).

(39) $$n_{total}=n_{excess}+C_{gas,eq}V_{void},$$

In any case, it is necessary to calculate void volume. This parameter can be calculated by helium picnometry by assuming that helium is neither adsorbed nor absorbed in the porous solid and that it does not penetrate regions that are inaccessible for the sorptive gas. This is not always the case, especially for microporous solids. Therefore, other techniques such as difference measurements or a no void analysis (NOVA) can be conducted [77].

Materials for gas adsorption are paramount in applications like the refining of biogas to bio-methane, hydrogen or methane storage [111], air separation into N_2_ and O_2_, separation of syngas into CO and H_2,_ or the removal of impurities (e.g., air drying or air pollution) [15]. These applications require activated carbons with large particle sizes (e.g., granules or pellets) and large bulk densities to avoid large pressures drops. For this reason, some chemically activated carbons are not suitable for these applications. To overcome this problem, the powders can be pelletized or granulized to obtain a larger particle size [112]. Another possible solution is to shape the activated carbons into honeycombs or other types of monoliths [112,113,114]. 

### 4.4. Adsorption in Liquid Media

**Figure 6:**
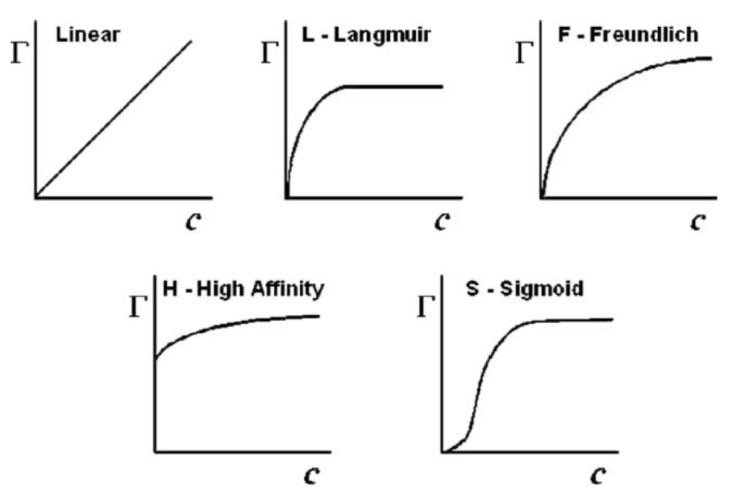
Isotherm models for adsorption in liquid media (reprinted from [116] with permission from Elsevier).

Adsorption in liquid media is extremely relevant in applications like removal from organic compounds or heavy metals in sewage systems or industrial water treatments. It is also commonly used in the food industry to remove compounds that give undesired color or odor to the final product. Like the adsorption of gases, adsorption in solutions can also be described by means of isotherms (Figure 6). These isotherms are a mathematical relation between the amount of adsorbate adsorbed by 1 g of adsorbent and the solute concentration after equilibrium has been reached at constant temperature. The most common models used to describe different isotherms are the ones proposed by Langmuir (Equation (40)) and Freundlich (Equation (41). As it was previously mentioned, the Langmuir model is the basis for many of the models presented already and it assumes that the adsorbent can only be covered by a monolayer of the adsorbate, excluding the possibility of a multilayer formation. Freundlich proposed a logarithmic change with the intention of including adsorbate-adsorbate interaction and, hence, considering a multilayer adsorption.

(40) $$q=\frac{q_{m}KC}{1+KC},$$

(41) $$q=kC^{\frac{1}{n}},$$

where *q* is the amount adsorbate adsorbed per mass unit of adsorbent, *q_m_* is the maximum mass adsorbed per mass unit of adsorbent, *C* is the concentration at equilibrium (it is worth noting that concentration *C* is interchangeable with pressure, if the adsorbate is a gas instead of a dissolved substance), *K* is the Langmuir constant related to the adsorption heat, *k* is the Freundlich constant related with adsorption capacity of the adsorbent and n is the adsorption intensity [115]. Prausnitz-Radke proposed a model, where both Langmuir (if *β* = 1) and Freundlich (if *bC^β^* >> 1) behaviors are included (Equation (42)):

(42) $$q=\frac{a_{PR}C}{1+b_{PR}C^{\beta }},$$

where *a_PR_*, *b_PR_* and *β* are constants. Temkin suggested with his model that adsorption enthalpy increases with increasing surface coverage, suggesting that the most energetic sites are occupied first (Equation (43)).

(43) $$q=a_{T}ln\left(b_{T}C\right),$$

where *a_T_* and *b_T_* are constants related to the linear change of the adsorption enthalpy as a function of the concentration.

The isotherm shape depends on the adsorption mechanism, which in turn is governed by the textural properties of the activated carbon, the surface chemistry (surface groups and *pH_PZC_*), the adsorbate properties (molecular size, polarity, solubility and concentration), and the solution properties (pH, temperature and ionic strength).

**Figure 7:**
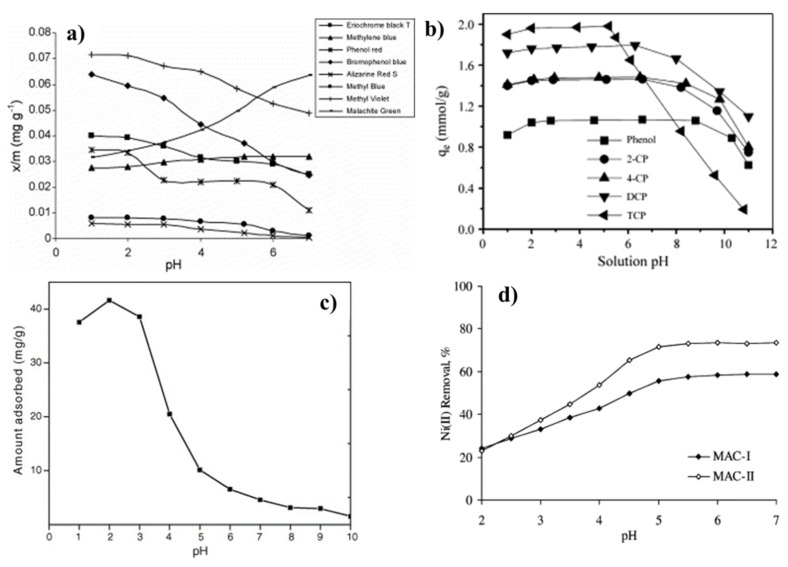
pH dependence of the adsorption of (**a**) different dyes (**b**) different phenolic compounds: 2-chlorophenol (2-CP), 4-chlorophenol (4-CP), 2,4-dichlorophenol (DCP), 2,4,6-trichlorophenol (TCP), (**c**) Cr(IV), (**d**) Ni(II) (adapted from [120,121,122,123] with permission from Elsevier).

When a porous solid comes in contact with an aqueous solution, a charge distribution on the surface is formed because of interactions between the surface groups and the solution ions (electrochemical double layer). This charge distribution depends strongly on the solution pH and the surface net charge, which can be determined by the *pH_PZC_* and by the isoelectric point (IEP). According to J. Menéndez et al., IEP gives information on the external surface charges and the *pH_PZC_* is a response of the total surface charges [117]. The *pH_PZC_* can be determined by acid/base or mass titration methods and is defined as the pH at which the net surface charge is zero [118,119]. This means that the amount of H+ ions adsorbed on the surface is the same as that of OH− ions; thus the charge on the surface is neutral. Since the surface charge is a pH function, this parameter becomes extremely relevant in adsorption processes in liquid media. When immersed in an electrolyte solution, the adsorbent will be surrounded by ions with an opposite charge as that of the surface. This has a direct influence on the charge distribution of the surface, which in turn influences the electrostatic interactions between adsorbent and adsorbate as well as the adsorption isotherm. Depending on the acidic nature of the surface, conducting adsorption in a solution with a low pH can either promote or impede adsorption of a certain species. Figure 7 shows some examples of the pH dependence for the adsorption of different compounds. Organic compound adsorption such as dyes and phenol-like substances is promoted by lower pH values, and heavy metals are adsorbed better, either in acid or slightly neutral solutions. Of course, these examples are by no means general tendencies and it is not possible to state that they are an absolute. The reason is that, if the surface chemistry of the adsorbent is modified, the solution pH at which the maximum adsorption potential occurs, will also change.

It should be noted that to remove contaminants in liquid media, a large surface area is not necessarily a determinant factor. Hydrochars have very low surface areas ranging between 0.5 to 30 m^2^/g but despite of this, they can remove different substances like congo red, 2-napthol, pyrene, acetaminophen and other organic micropollutants or heavy metals [124,125,126,127,128]. The most probable explanation to why hydrochars are good adsorbents for polar substances are the aryl and alkyl moieties of the amorphous carbon structures [126].

Adsorption in aqueous media can also be employed to texturally characterize activated carbons. One example is adsorption of iodine, also known as iodine number. This technique has been widely employed as a fast test to obtain information regarding the internal surface area of a porous material [129]. The adsorption mechanism of iodine is like that of N_2_ at 77 K: iodine enters micropores by pore filling and mesopores by capillary condensation [130]. Even though the adsorption mechanisms are similar, there is no direct correlation between BET surface areas calculated from N_2_ and CO_2_ isotherms and the iodine number. On the other hand, iodine number is a convenient parameter to obtain trends and for comparative purposes. Other compounds commonly used for quality tests are methylene blue and phenol. The methylene blue index gives information on the activated carbon capacity to adsorb voluminous molecules and is usually used to test activated carbons that will be employed in medicinal applications. The phenol index is a common parameter for activated carbons that are employed for water treatment purposes [2].

## 5. Other Characterization Techniques

For adsorption to occur, the reaction enthalpy *ΔG* must be negative. In addition, since adsorption usually takes place at relatively low temperatures, the entropy term *ΔS* can be neglected. Based on these assumptions and on Equation (44), it can be concluded that adsorption is an exothermic process (*ΔH* < 0; the exception to an exothermic adsorption process is when dissociation of a molecule occurs. In this case, it can be endothermic). This exothermicity is used sometimes to characterize adsorption processes by means of calorimetry.

(44)  ΔH=ΔG+TΔS<0 

To separate compounds from a solution, the adsorbent should have a network of micropores of different sizes to retain molecules. Additionally, meso- and macropores are necessary to allow access in the micropores. The amount of heat released during adsorption is proportional to the area covered by the adsorbate and this can be measured in liquid and gas systems with the help of immersion and adsorption calorimetry, respectively.

**Figure 8:**
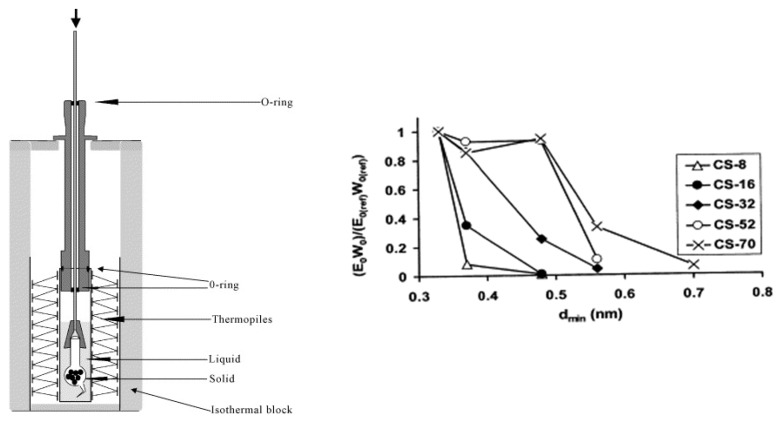
Experimental setup for immersion calorimetry (**left**; [131]) and pore size distribution obtained from immersion calorimetry measurement on different activated carbons using water, benzene, 2,2 dimethylbutane and iso-octane (**right**; [132]). Reprinted with permission from Elsevier.

Immersion calorimetry aims to describe adsorption in liquid/solid interfaces. Probably the first scientist to explore this methodology was M.C.S Pouillet, who discovered that immersing sand in water was an exothermic process [95]. The experimental setup for measuring immersion enthalpy usually consists of a well-isolated block in which a set of thermopiles surround the vessel that contains the probe liquid (Figure 8—left). Additionally, a previously degassed porous material is contained in a glass cell with a fragile tip that has been sealed under vacuum. The fragile tip is intended for reducing the energy contributions related to the breaking process and the vacuum supports the wetting process. After thermal equilibrium is reached, the glass cell is broken and temperature changes inside the system are recorded. A way to use immersion calorimetry to describe the internal surface area of activated carbons is acknowledging that the immersion energy is directly proportional to the area accessible to the probe molecule. For this reason, probe molecules with different sizes can be used to describe the micropore distribution (Figure 8—right).

The energy change during physisorption of gases is also known as differential heat of adsorption or isosteric heat. This enthalpy is the amount of energy released during the adsorption of a certain amount of gas or vapor on the surface of a porous solid and it is described by Equation (45).

(45) $$\left(\frac{\partial lnp}{\partial T}\right)=\frac{\Delta H}{RT^{2}},$$

where *ΔH* is the isosteric heat and *p* and *T* are the system pressure and temperature at equilibrium conditions. This equation is analogue to the Clausius-Clapeyron equation and can be integrated, with some approximations, as shown in Equation (46).

(46) $$\;\Delta H_{ads\;}=\frac{RT_{1}T_{2}}{T_{2}-T_{1}}ln\left(\frac{p_{2}}{p_{1}}\right)$$

From Equation (46), it is evident that at least two different states at equilibrium are required to calculate the isosteric heat of adsorption. For this reason, it is necessary to measure isotherms at different temperatures. The points corresponding to a certain amount of adsorbate adsorbed per unit of mass are plotted in a *ln p* vs. 1/*T* graph, which results in a linear curve (adsorption isosteres). The isostere slopes correspond to the heats of adsorption for a certain surface coverage. 

## 6. Microporous Carbon Materials in Energy Storage Systems

Microporous carbon materials are frequently designated as activated carbons and related to adsorption applications. Due to its large surface areas and large micropore volumes, microporous carbon materials also play a critical role in energy storage. Currently, the carbon materials used in electrodes are fossil-based: activated carbon from fossil coal, glassy carbons from resins of phenol–formaldehyde or furfuryl alcohol–phenol or nanostructures like carbon nanotubes, fullerenes or nanofibers produced by means of arc discharge, laser ablation or chemical vapor deposition of different hydrocarbons [133]. In general, carbon materials for energy storage purposes have low bulk density, large surface area-to-volume ratio, high electrical conductivity, and are chemically and thermally stable. Additionally, the presence of heteroatoms is desired to increase pseudo-capacitive phenomena that result from fast and reversible redox reactions and from Faradaic charge transfer reactions. Recent research has shown that it is possible to develop bio-based carbon materials with properties similar or superior to their fossil-based counterpart in galvanic elements such as lithium (LIB) or sodium-ion batteries (SIB), supercapacitors, microbial fuel cells [134,135] or fuel cells [136,137,138].

Electrochemical double-layer capacitors (also known as ultra or supercapacitors) store energy electrostatically due to reversible ion adsorption on the active material. This creates a double layer on the electrode-electrolyte interface. The active material in both electrodes of a supercapacitor is usually a highly porous carbon since the energy stored is directly proportional to the available surface area A. Other variables that affect energy storage are the dielectric constant of the material *ɛ_r_* and of vacuum *ɛ_0_*, and it is inversely proportional to the thickness of the double layer *d* (Equation (47)).

(47) $$\;E=\frac{\varepsilon_{0}\varepsilon_{r}A\;}{d}$$

The carbon materials used in electrodes can be doped with heteroatoms, metal oxides, or metal nitrides to increase faradaic effects. A supercapacitor with aqueous electrolyte is said to be asymmetric if the positive electrode is made with these pseudocapacitive materials (the term hybrid supercapacitor is usually used for supercapacitors that work with organic electrolytes). Asymmetric supercapacitors are designed to increase the energy density and cell voltage, but the cycling life is shorter because of the active material degradation [139]. Research on biomass as precursor to producing biobased carbon materials, with and without doping agents, for supercapacitors has increased drastically in the last 10 years. A quick search in Scopus shows that in 2007 merely 4 papers were published that included the words *biomass* and *supercapacitors*, whereas in 2017 almost 1700 original scientific articles and reviews were published. This indicates that biobased carbon materials have great potential to replacing their fossil-based counterpart and part of it is due to their versatility, environmental sustainability, and availability. The natural structure of biomass gives rise to carbon materials with interesting hierarchical organization and patterns that otherwise can only be achieved with templates [140,141,142]. Besides the textural properties that can be obtained with biomass, the introduction of functional groups in the carbon materials has been widely considered. In this case, techniques like *pH_PZC_* give valuable information concerning the adsorption of charges and reversible redox reactions on the carbon surface, which in turn provides some insight on the pseudocapacitive response of the carbons in different electrolytes [143,144,145].

**Figure 9:**
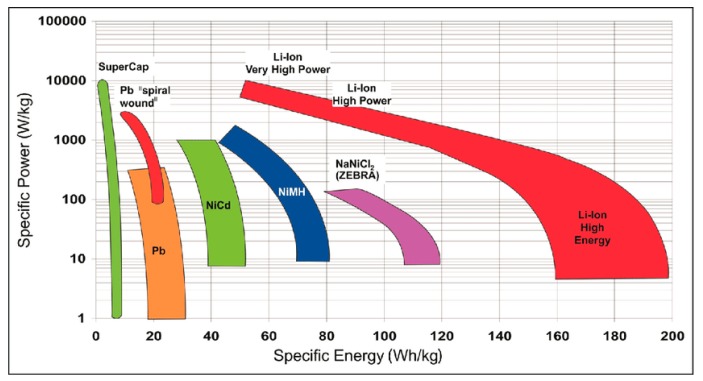
Ragone plot of different energy storage systems (reproduced with kind permission from [146]).

Increasing research interest on LIBs and SIBs has also been observed. Contrary to supercapacitors, LIBs and SIBs store energy electrochemically. The Ragone plot presented in Figure 9 shows the main differences in terms of specific power and specific energy between the main energy storage systems. Carbon materials in lithium or sodium-ion batteries play an important role as materials for negative electrodes. Lithium-ion batteries have become a staple system in the development of a CO_2_-neutral economy, since they can be used in a large range of energy storage applications—from assisting the electrical grid to small portable devices. These batteries have a low weight (lithium is the lightest metal) and it is possible to obtain high energy and power densities resulting from the strong electropositive character of lithium (E°_(Li+/Li)_ = −3.04 versus standard hydrogen electrode) compared to other alkali metals. Due to the reactivity of lithium with water, the positive electrode of the lithium-ion batteries is made of lithium oxides containing transition metals. On the other hand, the negative electrode is usually graphite or another form of carbon. 

Due to the rarity of lithium and its expensive processing, LIBs are considerably cost inefficient. Furthermore, life-cycle analyses have shown that electric cars using batteries can have a CO_2_ print as high as cars with internal combustion engines (especially if the energy to charge the battery is from fossil sources), since the battery production is very resource- and energy-intensive [147]. For this reason, the focus has started to shift towards the development of SIBs. SIBs were first introduced in the 1960s by N. Weber and J.T Kummer, who developed a system that worked at 300 °C using sodium and sulfur electrodes in the liquid phase and a solid beta-alumina electrolyte. [148]. Sodium (E°_(Na+/Na)_ = −2.71 versus standard hydrogen electrode) is more abundant and easily accessible than lithium and, being an alkali metal, its properties are comparable to those of lithium. The materials used for the positive electrode of SIBs are sodium oxides with different transition metals and, similar to LIBs, the negative electrode consists also of graphite or other carbon structures [149]. 

**Figure 10:**
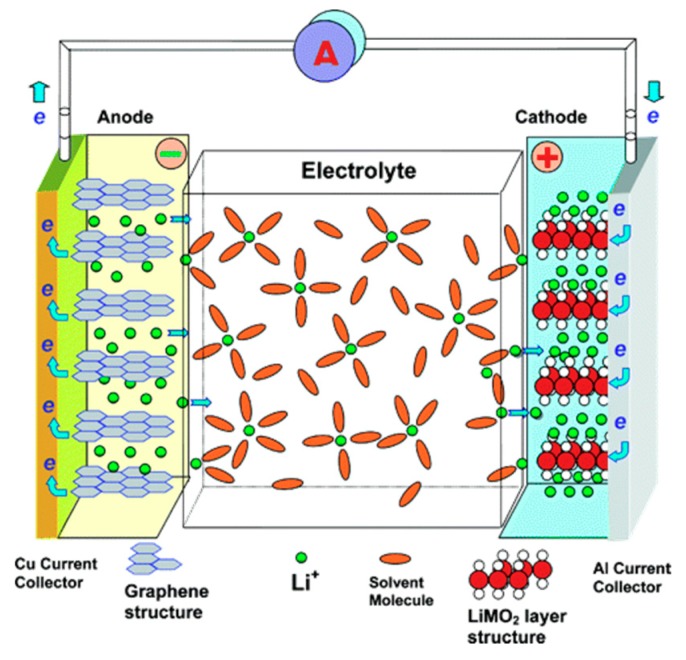
Scheme of a LIB showing the intercalation structure built between lithium and graphene sheets in the anode (reproduced with kind permission from [150]).

During charging, lithium ions are inserted between the graphene layers of graphite, building intercalation compounds with a maximum stoichiometric ratio of LiC_6_ and a theoretic capacity of 372 Ah/kg (Figure 10; in practice, the maximum capacity measured has been 335 Ah/kg for Li_0.9_C_6_) [150,151]. Due to its amphoteric character, the host carbon structure takes on a negative or a positive charge, respectively, when cations (e.g., Li^+^ or Na^+^) or anions (e.g., SO_4_^2−^ or Br^−^) are inserted in the lattice. For this reason, it can react at both high and low potentials. Amorphous carbon materials, also known as hard carbons, have a much less-organized structure than graphite and the cation intercalation in its structure as well as the capacity are strongly dependent on the textural properties of the carbon material and of the heterogeneous atoms present on the surface. S. Han et al. [22] studied the electrochemical properties of green tea leaves pyrolyzed between 700–900 °C for 2 h as well as the changes of potential caused by the adsorption/desorption of lithium ions. Increasing pyrolysis temperatures led to higher surface areas and pore volumes, but to smaller pore sizes. Consequently, carbons produced at lower temperatures presented more active sites for lithium ion insertion and higher diffusion rates; hence, these materials showed higher cyclic capacities and superior rate capabilities. F. Zheng et al. [152] did not only carbonize biomass at high temperatures, but also impregnated the carbons with HNO_3_ to increase the nitrogen functionalities on the surface. They also observed a positive effect of temperature on the surface area and pore volume, but the pore size remained almost constant. They measured a reversible capacity of 708 and 1071 mAh/g, which decreased to 630 mAh/g after 1000 cycles, which they attributed to the crystalline defects and pseudo-capacitive effects of the nitrogen atoms. The non-crystalline character of hard carbons is particularly important for SIBs, since the radius of sodium ions is considerably larger than that of lithium ions (0.102 nm vs. 0.076 nm), hence there are mass transport and storage limitations when using graphite [153]. Y. Zhang et al. prepared ordered mesoporous carbon materials doped with nitrogen groups using honey as a precursor and tested them as anodes for LIBs and SIBs [154]. The surface area obtained was 677 m^2^/g and the reversible capacities were as high as 1653 mAh/g for LIBs and 427 mAh/g for SIBs. I. Izanzar et al. [155] explored higher pyrolysis temperatures to convert date pulp and seeds into hard carbons for the negative electrodes of SIBs. They observed a positive influence on the reversible capacities, which varied between 200 and 300 mAh/g. The electrochemical properties of biomass-derived hard carbon for the anodes are promising; however, a drawback of these materials is the low Coulombic efficiencies due to the formation of a solid electrolyte interface [156]. To overcome this problematic, aspects like the electrolyte nature, reducing contact area between the electrolyte and the carbon material in the anode, restricting the voltage window allowed during cycling, or passivating the electrode must be considered [157]. Consequently, the design, development and optimization of biobased carbon materials for SIBs and LIBs is a wide-open investigation field.

## 7. Conclusions and Outlook

This review summarizes how biomass can be converted into biobased carbon materials with examples of applications where these can be employed. Additionally, it shows that it is possible to replace fossil sources with sustainable, environmentally friendly sources, such as agricultural and other residues to produce porous carbon materials. However, there are still some areas in which research is necessary. For example, there is an enormous knowledge gap in the intersection between biomass conversion technologies and the applications fields. It is necessary to develop conversion processes (in both laboratory and industrial scales) that lead to tailor-made bio-based carbon materials for each application. The last sections show that this is possible and that there is a vast potential for biobased carbon materials. However, collaborations between these two worlds are required. The most relevant example that comes to mind is the application of biobased carbon materials in electrochemical applications. To make electromobility possible, as well as free from fossil sources, collaborations that create synergies and complementary knowledge are necessary.

## Figures and Tables

**Table 1:** Temperature dependence for the formation of the intercalate compounds formed by potassium and carbon (from Ref. [55]).

Stoichiometric Composition	Temperature (°C)	Mole% of K	wt. % of K
C_8_K	250–318	11	29
C_24_K	356–420	4.0	12
C_36_K	420–487	2.7	8.3
C_48_K	479–508	2.0	6.4
C_60_K	Above 500	1.6	5.1

**Table 2:** Typical measuring conditions and applications for the gases employed in gas adsorption methods [78].

Gas	Temperature (K)	Vapor Pessure (kPa)	Cross-Sectional Area (nm^2^/molecule)	Application
N_2_	77.4	101.35	0.162	Surface area determination
Ar	87.3	101.35	0.142	Micropore analysis
CO_2_	273.1	3485.2	0.21	Pore size distribution for pores <1 nm
Kr	77	0.35	0.205	Materials with low surface areas
